# Supplementary material for: Role of the regulator in enabling a just culture: a qualitative study in mental health and hospital care
Source: BMJ Open. 2022 Jul 27;12(7):e061321. doi: 10.1136/bmjopen-2022-061321 (PMC9335042; doi:10.1136/bmjopen-2022-061321)
Supplement: Supplementary data [file bmjopen-2022-061321supp001.pdf]

Attachment A

Working definition of just culture in the project

A just culture is a culture of trust in which employees feel free to be open about insecurity and fallibility. In particular, the focus is on the behavior rather than the outcomes of that behavior, because behavior is something that healthcare professionals can control, while outcomes are partly dependent on factors outside the direct sphere of influence of the healthcare professional.

Learning and improving instead of punishment are paramount in a just culture. There is an open, safe and informal culture of approach, in which people work together on the quality of care in a continuous learning cycle. However, this does not mean that people within a just culture cannot be held responsible for their behavior. A distinction is made between ‘blaming and being punished' and 'accountability'.

People who are involved in undesirable outcomes or who have made mistakes are treated fairly and a balance is struck between accountability for a mistake made and learning and improving from that mistake. A mistake made is mainly seen as a reason to learn from within a just culture. System factors are also explicitly considered. The relevant question is: what caused this situation to arise? Peer support of 'second victims' is a natural part of a just culture, in addition to, of course, attention for primary victims.

A just culture recognizes that there is no single truth of an event. It is about valuing and considering multiple perspectives on an event. Justice means: do not judge from one perspective, but include as many perspectives as possible. This plurality requires a dialogue. Norms, such as those laid down in clinical guidelines, are considered here, but never used as a standard; attention is always paid to the situation from which action was taken and the interpretations of the standard that were used.

A central concept for a just culture is trust; trust among employees so they can also speak out to each other, trust of employees in managers that they are treated fairly, trust of patients and their families that they are treated with respect and that errors result in learning and improvement.
