# Supplementary material for: Role of the regulator in enabling a just culture: a qualitative study in mental health and hospital care
Source: BMJ Open. 2022 Jul 27;12(7):e061321. doi: 10.1136/bmjopen-2022-061321 (PMC9335042; doi:10.1136/bmjopen-2022-061321)
Supplement: Author's manuscript [file bmjopen-2022-061321.draft_revisions.pdf]

BMJ Open is committed to open peer review. As part of this commitment we make the peer review history of every article we publish publicly available.

When an article is published we post the peer reviewers' comments and the authors' responses online. We also post the versions of the paper that were used during peer review. These are the versions that the peer review comments apply to.

The versions of the paper that follow are the versions that were submitted during the peer review process. They are not the versions of record or the final published versions. They should not be cited or distributed as the published version of this manuscript.

BMJ Open is an open access journal and the full, final, typeset and author-corrected version of record of the manuscript is available on our site with no access controls, subscription charges or pay-per-view fees (http://bmjopen.bmj.com).

If you have any questions on BMJ Open's open peer review process please email info.bmjopen@bmj.com

BMJ Open

# The role of the regulator in enabling a just culture: a qualitative study in mental health and hospital care

| | |
|---|---|
| Journal: | *BMJ Open* |
| Manuscript ID | bmjopen-2022-061321 |
| Article Type: | Original research |
| Date Submitted by the Author: | 02-Feb-2022 |
| Complete List of Authors: | Weenink, J; Erasmus University Rotterdam<br>Wallenburg, Iris; Erasmus Universiteit Rotterdam, Erasmus School of Health Policy and Management<br>Hartman, Laura; Council for Public Health and Society; Netherlands Centre for Ethics and Health<br>van Baarle, Eva; Vrije Universiteit Amsterdam, Department of Medical Ethics, Law and Humanities; Netherlands Defence Academy<br>Leistikow, Ian; Erasmus University Rotterdam, Erasmus School of Health Policy and Management; Health and Youth Care Inspectorate<br>Widdershoven, Guy; Vrije Universiteit Amsterdam, Department of Medical Ethics, Law and Humanities<br>Bal, Roland ; Erasmus Universiteit Rotterdam Instituut Beleid en Management Gezondheidszorg, Erasmus School of Health Policy and Management |
| Keywords: | QUALITATIVE RESEARCH, Health & safety < HEALTH SERVICES ADMINISTRATION & MANAGEMENT, Quality in health care < HEALTH SERVICES ADMINISTRATION & MANAGEMENT |
| | |

SCHOLARONE™
Manuscripts

*I, the Submitting Author has the right to grant and does grant on behalf of all authors of the Work (as defined in the below author licence), an exclusive licence and/or a non-exclusive licence for contributions from authors who are: i) UK Crown employees; ii) where BMJ has agreed a CC-BY licence shall apply, and/or iii) in accordance with the terms applicable for US Federal Government officers or employees acting as part of their official duties; on a worldwide, perpetual, irrevocable, royalty-free basis to BMJ Publishing Group Ltd ("BMJ") its licensees and where the relevant Journal is co-owned by BMJ to the co-owners of the Journal, to publish the Work in this journal and any other BMJ products and to exploit all rights, as set out in our licence.*

*The Submitting Author accepts and understands that any supply made under these terms is made by BMJ to the Submitting Author unless you are acting as an employee on behalf of your employer or a postgraduate student of an affiliated institution which is paying any applicable article publishing charge ("APC") for Open Access articles. Where the Submitting Author wishes to make the Work available on an Open Access basis (and intends to pay the relevant APC), the terms of reuse of such Open Access shall be governed by a Creative Commons licence – details of these licences and which Creative Commons licence will apply to this Work are set out in our licence referred to above.*

*Other than as permitted in any relevant BMJ Author's Self Archiving Policies, I confirm this Work has not been accepted for publication elsewhere, is not being considered for publication elsewhere and does not duplicate material already published. I confirm all authors consent to publication of this Work and authorise the granting of this licence.*

For peer review only

# The role of the regulator in enabling a just culture: a qualitative study in mental health and hospital care

Jan-Willem Weenink [a,*], Iris Wallenburg [a], Laura Hartman [b,c], Eva van Baarle [d, e], Ian Leistikow [a,f], Guy Widdershoven [d], Roland Bal [a]

a. Erasmus School of Health Policy & Management, Erasmus University Rotterdam, the Netherlands
b. Council of Public Health & Society, The Hague, the Netherlands
c. The Netherlands Centre for Ethics and Health, The Hague, the Netherlands
d. Department of Medical Ethics, Law and Humanities, Amsterdam University Medical Centers, Vrije Universiteit, Amsterdam, the Netherlands
e. Netherlands Defence Academy, Breda, the Netherlands
f. Dutch Health and Youth Care Inspectorate, Ministry of Health, Welfare and Sport, Utrecht, the Netherlands

*** Corresponding author:**
Dr. Jan-Willem Weenink
P.O. Box 1738, 3000 DR Rotterdam, the Netherlands
Email: weenink@eshpm.eur.nl
Telephone: +31 10 408 8555
Fax: not applicable

**Key words**
Government regulation
Quality of Health Care
Patient safety
Health Facilities
Qualitative research

**Word count: 4288**

For peer review only

**Abstract**

**Objectives**: Just culture is considered a promising way to improve patient safety and working conditions in the healthcare sector, and as such is also of relevance to healthcare regulators that are tasked with monitoring and overseeing quality and safety of care. The objective of the current study is to explore the experiences in healthcare organizations regarding the role of the healthcare inspectorate in enabling a just culture.

**Design**: Qualitative study using interviews and focus groups that were transcribed verbatim, and observations of which written reports were made. All data were thematically analysed.

**Setting:** Three mental healthcare providers, two hospitals, and the healthcare inspectorate in the Netherlands

**Participants**: We conducted 61 interviews and 7 focus groups with healthcare professionals, managers and other staff in healthcare organizations and with inspectors. Additionally, 27 observations were conducted in healthcare organizations.

**Results:** We identified three themes in our data. First, professionals and managers in healthcare organizations perceive the inspectorate as a potential catalyst for learning processes, for example as an instigator of investigating incidents thoroughly, yet also as a potential barrier as its presence and procedures limit how open employees feel they can be. Second, just culture is considered relational and layered, meaning that relationships between different layers within or outside the organization might hinder or promote a just culture. Finally, for inspectors to enable a just culture requires finding a balance between allowing organizations the time to take responsibility for quality and safety issues, and timely regulatory intervention when healthcare providers are unwilling or unable to act.

**Conclusions:** If regulators intend to enable the development of a just culture within healthcare organisations, they must adopt regulatory procedures that support reflection and learning within the organisations they regulate and consider mutual trust as a vital regulatory tool.

**Strengths and limitations of the study**

- A strength of this study is the amount and variety of collected data. This rich qualitative dataset from two healthcare sectors enables us to understand processes of just culture and regulation from 'within', which is needed as most of the literature on just culture remains theoretical.
- Healthcare organizations were motivated to work on a just culture with the inspectorate. It is unknown whether studying organizations that are less motivated or less comfortable with the inspectorate would have resulted in additional insights about the role of regulation in enabling a just culture.
- We need to be careful to generalize findings across and within settings as the precise role of regulation and regulators might depend on context of a healthcare sector as well as the national context of regulation.



For peer review only - http://bmjopen.bmj.com/site/about/guidelines.xhtml

For peer review only

# Introduction

Standards and protocols as well as practices such as root-cause analysis have been instrumental in enhancing quality and safety of care. Increasingly though, criticisms are voiced about their inability to take into account the complexity of healthcare, urging that further improvements must be sought in culture and behavior.[1-4] Just culture has been proposed as a means to further enhance quality and safety of health care.[5]

The concept of just culture is not easily described and different meanings and conceptualisations exist in literature and healthcare practice. Reason introduced the concept as an attribute of a safe culture, which has resulted in flow-charts or culpability trees to determine whether a healthcare professional should be held accountable for a medical error.[6] Others have highlighted the emotional impact of medical errors and subsequent investigations on healthcare professionals and the need for restorative justice within a just culture.[7-9] A prospective focus on learning and healing are more central in this approach instead of a retrospective focus on understanding the error and whether individuals should be held accountable.[10] Finally, some conceptualise just culture as a culture in which employees feel free to speak up and voice concerns, not only after errors have occurred but whenever they feel the quality of care might be at risk.[11] These conceptualisations are not mutually exclusive and at the same time there are differences in focus and scope. Based on these conceptualisations though, we could consider openness and dialogue, and balancing accountability and learning and improving, as key characteristics of a just culture.

Because a just culture is expected to contribute to quality and safety of healthcare, the concept is also of relevance to healthcare regulators that are tasked with monitoring and overseeing quality and safety of care.[12, 13] Little is known however about how regulators can influence a just culture in healthcare organizations. The objective of the current study is to explore the role governmental regulation has regarding a just culture in healthcare organizations, and to reflect on what this means for policy and practice of healthcare regulators.

# Methods

## Setting

Our study focuses on regulation of healthcare in the Netherlands. The role of the Dutch Health and Youth Care Inspectorate (from now on: inspectorate) is to supervise quality and safety of both healthcare organizations and individual healthcare professionals.[14, 15] The inspectorate uses two approaches: incident-based supervision following incidents and complaints, and risk-based supervision focusing on specific themes or type of providers. Dutch healthcare organizations are mandated by law to report sentinel events (meaning unintended harm to patients that led to death or serious injury) to the inspectorate and share the investigation report with the inspectorate.[16] In recent years, the inspectorate has focused its policy on learning and improvement of healthcare professionals and organizations, and in this context the current project takes place.

## Study design

For a period of 2.5 years, we studied how five healthcare organizations and a project group of the inspectorate worked on enhancing a just culture in healthcare organizations. The project


For peer review only - http://bmjopen.bmj.com/site/about/guidelines.xhtml

underlying our study was initiated by the inspectorate with the aim of understanding what is needed for a just culture and how the inspectorate can contribute to this. We used qualitative research methods such as observations and interviews to explore experiences with working on a just culture and the relationship with regulation. Participating organizations (i.e. 3 mental healthcare providers and 2 hospitals) were recruited after a seminar of the inspectorate about just culture and each started their own project on working on a just culture. They each opted for a specific approach based on their perspective on what was urgent and feasible in their organization. Approaches differed between working on specific processes (e.g. incident investigations) to broader approaches on quality and safety policies in the organization. Simultaneously, a project group of the inspectorate held regular meetings to reflect on preliminary findings and their own role as inspectors in enabling a just culture. The goal of our study was to identify overarching themes related to the role of regulation in enabling a just culture. Table 1 describes the objectives and approaches of the five participating organizations and the inspectorate. In this paper, we specifically focus on the role of regulation and the regulator, not on the five organisational approaches of working on a just culture.

*Table 1: Description of objectives and initiatives to enable a just culture in participating mental healthcare (MH) and hospital (H) organizations and inspectorate*

| Organization | Objectives | Initiatives |
|---|---|---|
| MH1 | Strengthening the involvement, ownership and learning of employees in sentinel event investigations | Series of dialogue sessions with employees (managers, psychiatrists, psychologists, nurses and psychotherapists) aimed at exploring past experiences with sentinel event investigations, and a feedback session in which findings were discussed with all participating employees. |
| MH2 | Finding an appropriate approach to learn from sexual boundary violations and prevent such violations in the future | A "fishbowl session" in which two former patients (victims) and team members discussed the incidents and focused on what the team and the organization could learn from these incidents. Members of the management team observed without interrupting. |
| MH3 | Evaluating the organization's patient safety policy | There were established patient safety policies and initiatives, e.g. a safety café in which people share personal experiences about fallibility and learning. These policies and initiatives were discussed and reflected upon from a just culture perspective. |
| H1 | Improving sentinel event investigations | Workshops were organized for incident-investigators within the organization, aimed at fostering reflection and learning from current investigative strategies and at developing competences for writing more appreciative analysis reports. |
| H2 | Developing an approach to quality of care based on "learning from what goes well" and "personal involvement" | Weekly quality-meetings in which discharged patients and scheduled admissions and procedures were discussed. |
| Inspectorate | To understand how a just culture is enabled in organizations and what role regulation and inspectors have in this | Project group with inspectors from hospital and mental healthcare that organized meetings to reflect on preliminary findings during the study and interacted with participating organizations. |


For peer review only - http://bmjopen.bmj.com/site/about/guidelines.xhtml

**Data collection**

In preparation of the empirical research in the organizations, interviews were held with employees of the inspectorate to gain insight into the way inspectors interpret the concept of a just culture and how they view their own role. Subsequently, we observed meetings, held interviews and conducted focus groups in the participating organizations. The interviews and focus groups were recorded (audio) and transcribed, while written reports were made of the observations. During the project, we presented and reflected on preliminary findings within the organizations. In addition, we organized three network meetings. Here, representatives of the five organizations and the inspectorate came together and shared their experiences to learn from each other. At the end of the project, we organized three focus groups with inspectors in which we fed back the results from the organizations and reflected on what these findings mean for the regulator. In total, the data collected for this study consisted of 61 interviews, 7 focus groups and 27 observations. Table 2 provides an overview of all data collection methods.

*Table 2: Overview of data collection*

| **Location** | **Activities for data collection** |
|---|---|
| Inspectorate | ○ 8 interviews with inspectors to explore the concept of just culture and potential role of the inspectorate at the start of the project<br>○ 3 focus groups with inspectors (4-8 per group) to reflect on the findings at the end of the project |
| Mental healthcare organisation #1 | ○ 7 dialogue sessions with ±4 participants of different layers of the organization<br>○ 1 reflection session |
| Mental healthcare organisation #2 | ○ 2 dialogue sessions<br>○ 10 interviews with participants of dialogue sessions<br>○ 6 interviews with professionals<br>○ 2 interviews with management |
| Mental healthcare organisation #3 | ○ 17 interviews<br>○ 4 observations<br>○ 2 focus groups<br>○ 1 reflection session |
| Hospital #1 | ○ 11 interviews with 14 persons<br>○ 2 focus groups |
| Hospital #2 | ○ 7 interviews<br>○ 12 observations<br>○ 1 conference meeting |
| Network sessions | ○ 3 meetings with organizations and inspectorate aimed at exchanging experiences between participating organizations |


For peer review only - http://bmjopen.bmj.com/site/about/guidelines.xhtml

### Data analysis

The analysis focused on exploring overarching and recurring themes in the data.[17] The first interviews were inductively coded by involved researchers and aimed to identify common patterns in the data. These patterns included factors and mechanisms related to enabling a just culture in organizations in general, not on the role of regulation specifically. These patterns were then discussed, adjusted and further elaborated on during discussions with the research group, leading to a coding scheme. Subsequent transcripts of interviews and focus groups and notes of observations were coded using this scheme and new themes were added as they emerged. Findings from the transcripts, observations and meetings were discussed within the research group and fed back to participating organizations and inspectors throughout the project. To understand and reflect on the specific role of regulation in enabling a just culture, the findings were discussed again with the purpose of this study in mind, leading to three main themes related to regulation.

### Patient and public involvement

There was no patient or public involvement.

### Research Ethics

The Medical Research Ethics Committee of the Erasmus Medical Centre determined that the study did not fall within the scope of the Medical Research Involving Human Subjects Act and as such did not require additional ethical approval (MEC-2018-054). All respondents were verbally informed about the study and gave their approval for recording interviews and focus groups. To ensure anonymity, some details from quotations have been adjusted and we do not specify from which organizations quotations come.

## Findings

From our analysis, we identified three themes that are important to understand the role of governmental regulation in enabling a just culture in healthcare organizations: 1) regulatory impact on a just culture, 2) the relational and layered nature of a just culture, and 3) challenges for regulators and inspectors.

### 1. Regulatory impact on a Just Culture

When respondents elaborated on the role and impact of the regulator in enabling a just culture, they referred to issues regarding the image of the regulator and the rigidity of forms and procedures.

#### *Police or driver of quality improvement?*

Respondents perceive the inspectorate as a threat for creating a just culture, as they come into play when things already have gone wrong to judge about what has gone and has been done wrong. Although the inspectorate's scope and tasks are broader, this perception does affect the safety and openness that employees experience when trying to learn from incidents.


For peer review only - http://bmjopen.bmj.com/site/about/guidelines.xhtml

*"That's how I see the inspectorate. When they come to the hospital, something is going on somewhere. They don't just show up, you know. The police also does not come to your house for a cup of coffee. Then there is something going on as well. That's how I see it."*

There is a perceived threat among professionals of being held responsible for (their share in) an incident, and that being open can backfire. Inspectors recognize this tension yet refer to the professional standards that healthcare professionals must adhere to. According to inspectors, not calling on individual responsibility is difficult when sentinel events involve culpable personal actions of a professional.

At the same time, the inspectorate may act as an important driver for quality improvement. The image of the inspectorate and possible measures they might take ensure that healthcare organizations take sentinel event investigations seriously. They want to do it thoroughly and make time and resources available for it.

*"And well, then we found out that the medication used is already off the market in various hospitals. You just go deeper, deeper and deeper because of that investigation. I just wonder if the mandated investigation [of the inspectorate] had not been there, would we have gone that deep?"*

The inspectorate's image thus not only has negative consequences, but also implies authority that leads to action in organizations to improve patient safety.

***Rigid forms and procedures when things have gone wrong***

Although the inspectorate might be a catalyst for thorough investigative processes, this does not directly mean that these processes also contribute to learning among healthcare professionals. Respondents indicated that the tight timetable with hard deadlines for investigating and reporting sentinel events, in combination with the length of such reports, frustrate openness and thus learning. It means that there is limited space to reflect, and although reflection should be part of the investigation, it is not always experienced as such. There is a risk that meeting the inspectorate's requirements gains priority over the learning process:

*"There is a time limit of 8 weeks, then it takes time before you get [the report] back from the inspectorate. And you have a time limit in which [a sentinel event] must be reported. So, you cannot just think calmly whether or not to report [the event], whether to investigate."*

Respondents emphasize the importance of properly recording everything for the investigation. They perceive that what is written down on paper is more important than what exactly happened in practice. This 'paper-based reality', in which the focus is mainly on factual matters in combination with the formal language used in the report, are insufficiently in line with how professionals perceive their work. Consequently, professionals sometimes feel distanced from the reporting.

*"It is a business-like format that mainly looks at factual summaries of things that have been discussed. (...) People do perceive that [the inspectorate] finds it more important that everything*


For peer review only - http://bmjopen.bmj.com/site/about/guidelines.xhtml

> *on paper is correct instead of the actual care we provided. Because that story is almost deleted from those documents. (...) So, in the team you see that when the report is finally done, that people have a bit of a hangover from [the investigation report].”*

Some inspectors acknowledge the need for available instruments and procedures to match the goal of learning and improving and recognize that current forms and procedures do not facilitate such an approach.

> *“I also think that if we want to get a just culture into our DNA from our actions, then we need to carefully examine the systems we work with, the forms we work with and the questions we ask and see whether they are just culture-proof and are focused on learning and prevention in the future.”*

This requires the inspectorate to reflect on their own procedures from such a learning perspective.

**2. The relational and layered nature of a just culture**

Just culture was considered relational and layered by respondents, meaning that relationships between actors from different layers within and outside the healthcare organization might hinder or promote a just culture.

***Building relationships of mutual trust***

Respondents from the organizations reported that a just culture relies on mutual trust. This applies to different actors within the healthcare organization, for example between professionals, superiors and management, but also in relation to the inspectorate. This means that the feeling that employees get from the inspectorate and individual inspectors is important for experiencing a just culture. Their feeling during inspections is influenced by the procedures and correspondence of the inspectorate.

> *“You wait for some sort of grade from your schoolteacher, it always feels like that. While you would like much more dialogue at the table, ‘what do we learn from this’? That the inspectorate thus gains a sense of the learning capacity of an organization from their supervisory role, and not through letters with reference numbers, on which we then disagree and what results in writing another letter back.”*

Building trust is also about being open about regulatory procedures as an inspector, without perhaps always being able to offer a safe environment. This procedural clarity is seen by inspectors as part of a just culture, in which the quality and safety of patient care must come first. It means that inspectors must be clear that they cannot guarantee that someone will not be held personally accountable in case of sentinel events but that it is very unlikely. Inspectors notice that this openness and transparency about procedures on their part – in general or specifically when an incident has occurred – contributes to understanding and trust, and respondents from organizations experience it positively when the inspectorate elaborates and explains their procedures and its position.



For peer review only - http://bmjopen.bmj.com/site/about/guidelines.xhtml

*"Two inspectors visited the staff and indicated what their working method is and how they view our hospital. And that is quite enlightening. We perceive them as a very annoying organization that is trying to catch us, but in reality, it's not that bad. [Our staff] suddenly sees a face of these people, instead of just their firm notes and letters."*

Recognizing and building on the relationality of regulation is of importance in enabling a just culture as it helps in building trust and being able to talk about vulnerabilities.

***The role of publicity and legislation***

A factor that according to respondents influences openness, and thus learning, is publicity. While openness about an incident or sentinel event within the team or organization is seen by respondents as an essential component of a just culture, external publication and publicity pose a threat to it. Healthcare professionals perceive that anything they say might become public at some point, either via reports of the inspectorate or via the media. And although the inspectorate does not publish investigation reports with names of professionals, some professionals mention that even anonymized data are easily traceable. This perceived threat sometimes leads to openness and learning being disrupted, whereby what is written down on paper is again seen as important. It is more about hedging against any negative consequences of an investigation report, than about learning and improving. It could be more worthwhile to sit down and talk and reflect without writing things down on paper, as one of the healthcare professionals said:

*"I think so, if we lock ourselves up in a room for one afternoon, then there would just come out more. Things that are not written on paper and that do not go to the [national newspaper]. We would then learn even more from [the sentinel event]."*

Inspectors recognize the disrupting influence of media and other external actors on a just culture and have to deal with such influences themselves too. They experience that pressure from the media can lead to a feeling of insecurity among healthcare professionals.

In addition to potential publicity, respondents also mentioned the inhibiting nature of existing legislation and risks of litigation. Even though the inspectorate might adopt a learning perspective in their regulation, existing legislation on disciplinary complaints still focuses on individual accountability. Patients, for example, may choose to file a complaint against individual healthcare professionals.

*"There is also the disciplinary judge who is breathing down your neck. We are talking about a just culture, but how open is it when the threat of litigation lingers in the background?"*

**3. Challenges for regulators and inspectors**

Respondents, and specifically inspectors, mentioned several challenges for the regulator when trying to contribute to a just culture in healthcare organizations.


For peer review only - http://bmjopen.bmj.com/site/about/guidelines.xhtml

***Assessing a just culture***

Inspectors struggle with the question how to assess whether an organization has a just culture. Some inspectors indicate that instruments are available to get a feel for this, such as inspection frameworks on Trust and on Good Governance, in which openness, transparency and trust are important components. At the same time, inspectors realize that a just culture cannot be ticked off and that it is also about intuition and how confident you are that an organization itself is able to improve.

> *"Yes, a gut-feeling. When you are present at the administrative levels, then you need to understand the matter. So you ask the right questions to get a feeling of the organization. If that feeling is not good, then you should take a look at certain indicators."*

According to inspectors, asking questions implies a different attitude or style than a controlling one during an inspection visit. At the same time, as an inspector you never only act as a coach and discussion partner.

> *"We are also assigned a role depending on the situation. So, at one moment it is nice to be a discussion partner and at the other moment an organization needs you as a bogeyman to create urgency."*

The inspector cited above emphasizes the two roles inspectors can assume and that are expected of them, and the importance of finding a balance between giving space and keeping a firm hand on the healthcare provider. This means an inspector must be able to do both and must be able to sense which approach is necessary for a given situation.

***Informal contact and formal measures***

The fact that the inspectorate can impose sanctions can be at odds with the promotion of a just culture within healthcare organizations. According to inspectors, this makes it difficult because tensions arise between the space that an inspector sometimes wants to give to an organization to learn and improve (without formal interference from the inspectorate) and the policies and rules that prescribe certain sanctions, such as a monetary fine. Inspectors especially struggle when they have really invested in the relationship with an organization to ensure that the organization takes responsibility for quality and safety.

> *"I am the contact person for this organization. I went through this whole process with them and they clearly learned. It feels wrong to give them a fine at the end of this when they have come up with solutions themselves. I think that in our cooperative relationship, which I understand is a special kind of relationship, that's not good."*

Individual inspectors sometimes give a bit of space to organizations because they feel that this contributes to learning within the organization. Yet when formally judging on an organization, which is also the inspectorate's task (including possible measures), the case is discussed within the inspectorate. Sometimes there are different perspectives on what should be done between the involved inspector and other inspectors, managers and the legal department. This makes it

For peer review only

difficult, because for a healthcare organization it might seem as if there is a lot of space to learn and improve and figure things out whereas at the end of the process the organization might be confronted with an intervention from the inspectorate. So, although individual inspectors sometimes seem to have an eye for a just culture within the healthcare organization to facilitate learning, they are aware that trust in the relationship is fragile.

## Discussion

We explored the role of governmental regulation in enabling a just culture in healthcare organizations. Our results show that the regulator through its procedures and interaction with organizations has impact on learning processes and openness. Building mutual trust, for example by being clear about regulatory procedures and expectations, is deemed important, while publicity and external transparency might frustrate learning and openness. Finally, our study highlights challenges for regulators when it comes to assessing a just culture and the impact of legislation. We first provide a brief methodological reflection before we reflect on these findings.

### Strengths and limitations

A strength of this study is the amount and variety of collected data. This rich qualitative dataset from two healthcare sectors enables us to understand processes of just culture and regulation from 'within', which is needed as most of the literature on just culture remains theoretical.[7, 18] The study has some limitations as well. First, healthcare organizations were recruited after a seminar of the inspectorate and as such were actively interested in working on a just culture with the inspectorate. It is unknown whether studying organizations that are less motivated or less comfortable with the inspectorate would have resulted in additional insights about the role of regulation in enabling a just culture. Second, we need to be careful to generalize these findings across and within settings as the precise role of regulation and regulators might depend on context of a healthcare sector as well as the national context of regulation.

### Two issues for regulators when enabling a just culture

For regulators, two different issues seem important when aiming to enable a just culture. The first is the impact of regulatory procedures and actions on a just culture in healthcare organizations. As our study showed, the relation between the regulator and healthcare organizations and different people involved, influences the space for openness, reflection and learning in healthcare organizations. It requires reflection from regulators on their policies and procedures, and an understanding of how they directly impact (either positively or negatively) the reflective space in organizations.[2] A second issue is just culture as a focus of regulation itself. Inspectors felt the need to be able to assess whether an organization has a just culture. Although in international contexts tools have been developed to assess a just culture,[19] inspectors indicated that it requires intuition or a gut-feeling and that a just culture cannot simply be ticked off. It thus seems important that when choosing to use an assessment tool, it is used for inspectors to get a better understanding of an organization by combining it with other forms of soft and hard intelligence, instead of directly actioning regulatory measures based on the outcomes of the assessment.[20, 21] The latter most likely will not lead to an


For peer review only - http://bmjopen.bmj.com/site/about/guidelines.xhtml

organization working towards an open and learning culture but to an organization trying to score best on the measurements included in the assessment, risking to 'hit the target but miss the point'.[22]

**Balancing conflicting styles as an inspector**

Regulators are not an independent observer in monitoring quality and safety, but part of the healthcare playing field. Their actions and procedures influence practices within healthcare organizations. In our study this was apparent in the mentioned actions and approaches of inspectors through coaching or more policing styles towards healthcare organizations. These styles are inherent to responsive regulation theory describing persuasive and coercive enforcement styles, and which assumes that regulators should start with persuasive strategies before considering coercive ones.[23] These styles conflict and are not strictly successive as theory suggests, for example when expecting openness from professionals aimed at learning and at the same time keeping the possibility to file a formal complaint against an individual professional. The scope of the Dutch healthcare inspectorate – regulating both organizations and individual professionals – makes this conflict even more complex. At the same time, adopting different styles makes that healthcare organizations take inspectors seriously, as they can adjust to the specific needs of an organization. For inspectors, it is important to communicate the intentions of regulation and to continuously reflect on how to balance these styles in practice.[24] A further question for regulators would then be whether all inspectors should be able to conduct both styles or whether these styles are represented by different inspectors. The former would require different and for some inspectors new skills, whereas the latter would possibly create tensions between inspectors focused on learning and inspectors focused on policing.[25]

**Beyond the vacuum: taking third parties into account**

A just culture requires psychological safety in healthcare organizations, but also in the relation between regulator and healthcare organization.[26, 27] What is challenging is that this relation does not exist in a vacuum, and in enabling a just culture this is especially problematic when things have gone wrong. Often, patients and patient bodies, politics and media, quite understandably, demand thorough investigations, partly substantiated by a concern that certain things will otherwise be kept under the table. We've seen many examples in the past where these concerns were warranted.[28] The involvement of these other parties also means that the incident and subsequent investigation is taken outside the relation of regulator and healthcare organization, and the publicity and attention influences openness and learning within the organization. [29] This is something inspectors are aware of and that poses an additional challenge when trying to enable a just culture as a regulator. Being transparent about regulatory procedures and intentions towards those other parties might contribute to lowering the temperature of heated public discussions and as such contribute to the psychological safety of those involved. For healthcare organizations, directly involving patients or their representatives might contribute to trust and being able to investigate and learn out of the public spotlight.[30]

**Conclusion**

For peer review only

The regulator can have an important influence on a just culture in healthcare organizations. For regulators to be able to contribute to a just culture, it seems important to 1) be aware of the impact regulation and other stakeholders and policies have on a just culture, 2) adopt regulatory procedures that support reflection and learning in organizations, and 3) continuously reflect on how to balance more coaching and policing styles as inspectors. By doing so, regulators can contribute to learning within healthcare and as such improve quality and patient safety.

**Contributorship statement**

The study was designed by IW, LH, GW and RB, data was collected and analyzed by all authors, a first draft of the paper was written by JWW. All authors contributed to and approved the final manuscript.

**Competing interests**

None declared.

**Funding**

The study was funded by the Netherlands Organisation for Health Research and Development (ZonMw), project number 516004613.

**Data sharing statement**

No additional data are available.

**Ethics approval statement**

The Medical Research Ethics Committee of the Erasmus Medical Centre determined that the study did not fall within the scope of the Medical Research Involving Human Subjects Act and as such did not require additional ethical approval (MEC-2018-054).

For peer review only

## References


1. Leistikow I. The three stages of safety improvement. *The BMJ Opinion* blog. January 18, 2019, 2019.
2. Wiig S, Aase K, Bal R. Reflexive spaces: Leveraging resilience into healthcare regulation and management. *Journal of patient safety*. 2021;
3. Wears RL. Standardisation and its discontents. *Cognition, technology & work*. 2015;17(1):89-94.
4. Peerally MF, Carr S, Waring J, Dixon-Woods M. The problem with root cause analysis. *BMJ quality & safety*. 2017;26(5):417-422.
5. Dekker S. *Just culture: Balancing safety and accountability*. CRC Press; 2016.
6. Reason J. *Human error*. Cambridge university press; 1990.
7. Dekker SW, Breakey H. 'Just culture:'Improving safety by achieving substantive, procedural and restorative justice. *Safety science*. 2016;85:187-193.
8. Kaur M, De Boer RJ, Oates A, Rafferty J, Dekker S. Restorative just culture: a study of the practical and economic effects of implementing restorative justice in an NHS trust. EDP Sciences; 2019:01007.
9. White RM, Delacroix R. Second victim phenomenon: Is 'just culture'a reality? An integrative review. *Applied Nursing Research*. 2020;56:151319.
10. Sharpe VA. Promoting patient safety: an ethical basis for policy deliberation. *The Hastings Center Report*. 2003;33(5):S3.
11. Frankel AS, Leonard MW, Denham CR. Fair and just culture, team behavior, and leadership engagement: The tools to achieve high reliability. *Health services research*. 2006;41(4p2):1690-1709.
12. Marx D. Patient Safety and the Just Culture. *Obstetrics and gynecology clinics of North America*. 2019;46(2):239-245.
13. Weiner BJ, Hobgood C, Lewis MA. The meaning of justice in safety incident reporting. *Social science & medicine*. 2008;66(2):403-413.
14. Hout F, Nienhuis E, Robben P, Frederiks B. Supervision by the Dutch healthcare inspectorate. *Eur J Health L*. 2010;17:347.
15. Weenink J-W, Wallenburg I, Leistikow I, Bal RA. Publication of inspection frameworks: a qualitative study exploring the impact on quality improvement and regulation in three healthcare settings. *BMJ Quality & Safety*. 2021;30(10):804-811.
16. de Kam D, Kok J, Grit K, Leistikow I, Vlemminx M, Bal R. How incident reporting systems can stimulate social and participative learning: A mixed-methods study. *Health Policy*. 2020;124(8):834-841.
17. Braun V, Clarke V. *Successful qualitative research: A practical guide for beginners*. sage; 2013.
18. O'Donovan R, Ward M, De Brún A, McAuliffe E. Safety culture in health care teams: A narrative review of the literature. *Journal of Nursing Management*. 2019;27(5):871-883.
19. Petschonek S, Burlison J, Cross C, et al. Development of the Just Culture Assessment Tool (JCAT): Measuring the perceptions of healthcare professionals in hospitals. *Journal of patient safety*. 2013;9(4):190.
20. Kok J, Wallenburg I, Leistikow I, Bal R. The doctor was rude, the toilets are dirty. Utilizing 'soft signals' in the regulation of patient safety. *Safety Science*. 2020;131:104914.
21. Martin GP, McKee L, Dixon-Woods M. Beyond metrics? Utilizing 'soft intelligence' for healthcare quality and safety. *Social Science & Medicine*. 2015;142:19-26.
22. Radnor Z. Hitting the target and missing the point? Developing an understanding of organizational gaming. *Performance Information in the Public Sector*. Springer; 2008:94-105.


For peer review only

23. Ayres I, Braithwaite J. *Responsive regulation: Transcending the deregulation debate*. Oxford University Press, USA; 1992.
24. Mascini P, Wijk EV. Responsive regulation at the Dutch food and consumer product safety authority: An empirical assessment of assumptions underlying the theory. *Regulation & Governance*. 2009;3(1):27-47.
25. Furnival J, Walshe K, Boaden R. Emerging hybridity: comparing UK healthcare regulatory arrangements. *Journal of health organization and management*. 2017;
26. Khatri N, Brown GD, Hicks LL. From a blame culture to a just culture in health care. *Health care management review*. 2009;34(4):312-322.
27. Groeneweg J, Ter Mors E, Van Leeuwen E, Komen S. The long and winding road to a just culture. OnePetro; 2018:
28. Leape LL. A Conspiracy of Silence: Disclosure, Apology, and Restitution. *Making Healthcare Safe*. Springer; 2021:293-317.
29. Kok J. A standard story: On the use and consequences of standards in healthcare regulation. 2021;
30. Bouwman R, De Graaff B, De Beurs D, Van de Bovenkamp H, Leistikow I, Friele R. Involving patients and families in the analysis of suicides, suicide attempts, and other sentinel events in mental healthcare: a qualitative study in the Netherlands. *International journal of environmental research and public health*. 2018;15(6):1104.

For peer review only

BMJ Open

# The role of the regulator in enabling a just culture: a qualitative study in mental health and hospital care

| | |
|---|---|
| Journal: | *BMJ Open* |
| Manuscript ID | bmjopen-2022-061321.R1 |
| Article Type: | Original research |
| Date Submitted by the Author: | 29-Jun-2022 |
| Complete List of Authors: | Weenink, J; Erasmus University Rotterdam<br>Wallenburg, Iris; Erasmus Universiteit Rotterdam, Erasmus School of Health Policy and Management<br>Hartman, Laura; Council for Public Health and Society; Netherlands Centre for Ethics and Health<br>van Baarle, Eva; Vrije Universiteit Amsterdam, Department of Medical Ethics, Law and Humanities; Netherlands Defence Academy<br>Leistikow, Ian; Erasmus University Rotterdam, Erasmus School of Health Policy and Management; Health and Youth Care Inspectorate<br>Widdershoven, Guy; Vrije Universiteit Amsterdam, Department of Medical Ethics, Law and Humanities<br>Bal, Roland ; Erasmus Universiteit Rotterdam Instituut Beleid en Management Gezondheidszorg, Erasmus School of Health Policy and Management |
| <b>Primary Subject Heading</b>: | Health services research |
| Secondary Subject Heading: | Health policy, Qualitative research |
| Keywords: | QUALITATIVE RESEARCH, Health & safety < HEALTH SERVICES ADMINISTRATION & MANAGEMENT, Quality in health care < HEALTH SERVICES ADMINISTRATION & MANAGEMENT |
| | |

SCHOLARONE™
Manuscripts

*I, the Submitting Author has the right to grant and does grant on behalf of all authors of the Work (as defined in the below author licence), an exclusive licence and/or a non-exclusive licence for contributions from authors who are: i) UK Crown employees; ii) where BMJ has agreed a CC-BY licence shall apply, and/or iii) in accordance with the terms applicable for US Federal Government officers or employees acting as part of their official duties; on a worldwide, perpetual, irrevocable, royalty-free basis to BMJ Publishing Group Ltd ("BMJ") its licensees and where the relevant Journal is co-owned by BMJ to the co-owners of the Journal, to publish the Work in this journal and any other BMJ products and to exploit all rights, as set out in our licence.*

*The Submitting Author accepts and understands that any supply made under these terms is made by BMJ to the Submitting Author unless you are acting as an employee on behalf of your employer or a postgraduate student of an affiliated institution which is paying any applicable article publishing charge ("APC") for Open Access articles. Where the Submitting Author wishes to make the Work available on an Open Access basis (and intends to pay the relevant APC), the terms of reuse of such Open Access shall be governed by a Creative Commons licence – details of these licences and which Creative Commons licence will apply to this Work are set out in our licence referred to above.*

*Other than as permitted in any relevant BMJ Author's Self Archiving Policies, I confirm this Work has not been accepted for publication elsewhere, is not being considered for publication elsewhere and does not duplicate material already published. I confirm all authors consent to publication of this Work and authorise the granting of this licence.*

For peer review only

# The role of the regulator in enabling a just culture: a qualitative study in mental health and hospital care

Jan-Willem Weenink [a,*], Iris Wallenburg [a], Laura Hartman [b,c], Eva van Baarle [d, e], Ian Leistikow [a,f], Guy Widdershoven [d], Roland Bal [a]

a. Erasmus School of Health Policy & Management, Erasmus University Rotterdam, the Netherlands
b. Council of Public Health & Society, The Hague, the Netherlands
c. The Netherlands Centre for Ethics and Health, The Hague, the Netherlands
d. Department of Medical Ethics, Law and Humanities, Amsterdam University Medical Centers, Vrije Universiteit, Amsterdam, the Netherlands
e. Netherlands Defence Academy, Breda, the Netherlands
f. Dutch Health and Youth Care Inspectorate, Ministry of Health, Welfare and Sport, Utrecht, the Netherlands

*** Corresponding author:**
Dr. Jan-Willem Weenink
P.O. Box 1738, 3000 DR Rotterdam, the Netherlands
Email: weenink@eshpm.eur.nl
Telephone: +31 10 408 8555
Fax: not applicable

**Key words**
Government regulation
Quality of Health Care
Patient safety
Health Facilities
Qualitative research

**Word count: 4824**

For peer review only

**Abstract**

**Objectives**: Just culture is considered a promising way to improve patient safety and working conditions in the healthcare sector, and as such is also of relevance to healthcare regulators that are tasked with monitoring and overseeing quality and safety of care. The objective of the current study is to explore the experiences in healthcare organizations regarding the role of the healthcare inspectorate in enabling a just culture.

**Design**: Qualitative study using interviews and focus groups that were transcribed verbatim, and observations of which written reports were made. Transcripts and observation reports were thematically analysed.

**Setting:** Three mental healthcare providers, two hospitals, and the healthcare inspectorate in the Netherlands

**Participants**: We conducted 61 interviews and 7 focus groups with healthcare professionals, managers and other staff in healthcare organizations and with inspectors. Additionally, 27 observations were conducted in healthcare organizations.

**Results:** We identified three themes in our data. First, professionals and managers in healthcare organizations perceive the inspectorate as a potential catalyst for learning processes, for example as an instigator of investigating incidents thoroughly, yet also as a potential barrier as its presence and procedures limit how open employees feel they can be. Second, just culture is considered relational and layered, meaning that relationships between different layers within or outside the organization might hinder or promote a just culture. Finally, for inspectors to enable a just culture requires finding a balance between allowing organizations the time to take responsibility for quality and safety issues, and timely regulatory intervention when healthcare providers are unwilling or unable to act.

**Conclusions:** If regulators intend to enable the development of a just culture within healthcare organisations, they must adopt regulatory procedures that support reflection and learning within the organisations they regulate and consider mutual trust as a vital regulatory tool.

**Strengths and limitations of the study**

- A strength of this study is the amount and variety of collected data from two healthcare sectors.
- Participating organizations were motivated to work with the inspectorate in this study, whereas including organizations that are less motivated or less comfortable with the inspectorate could have resulted in additional insights.
- The study was conducted in one country, the Netherlands, whereas the precise role of regulation and regulators might depend on the national context of regulation.

For peer review only

## Introduction

Standards and protocols as well as practices such as root-cause analysis have been instrumental in enhancing quality and safety of care. Increasingly though, criticisms are voiced about their inability to take into account the complexity of healthcare, urging that further improvements must be sought in culture and behavior.[1-4] Just culture has been proposed as a means to further enhance quality and safety of health care.[5]

The concept of just culture is not easily described and different meanings and conceptualisations exist in literature and healthcare practice. Reason introduced the concept as an attribute of a safe culture, which has resulted in flow-charts or culpability trees to determine whether a healthcare professional should be held accountable for a medical error.[6] Others have highlighted the emotional impact of medical errors and subsequent investigations on healthcare professionals and the need for restorative justice within a just culture.[7-9] A prospective focus on learning and healing are more central in this approach instead of a retrospective focus on understanding the error and whether individuals should be held accountable.[10] Finally, some conceptualise just culture as a culture in which employees feel free to speak up and voice concerns, not only after errors have occurred but whenever they feel the quality of care might be at risk.[11] These conceptualisations are not mutually exclusive and at the same time there are differences in focus and scope. Based on these conceptualisations though, we could consider openness and dialogue, and balancing accountability and learning and improving, as key characteristics of a just culture.

The concept of just culture – albeit conceptualized in various ways – has been around for a few decades. Most papers on just culture in healthcare are of conceptual nature. [12-14] Empirical studies about just culture in healthcare remain limited.[15] Those that have been conducted focus on the impact of just culture training on the perceived organizational or safety culture,[16, 17] measuring tools for assessing a just culture,[18] what managers need in terms of personal competencies to effectively implement a just culture,[19] and on specific aspects of a just culture, such as peer support for second victims.[20]

Because a just culture is expected to contribute to quality and safety of healthcare, the concept is also of relevance to healthcare regulators that are tasked with monitoring and overseeing quality and safety of care.[21, 22] The role of regulation has been addressed in just culture literature. Dekker has called for the implementation of just culture in regulatory arenas and internationally there are examples of regulators that have implemented tools to regulate from a just culture perspective.[5, 23] Marx noted that 'regulators must become a force for error reduction rather than a force of error concealment'.[24] Little is known however about how regulators impact and could enable a just culture in healthcare organizations. The limited empirical work on just culture focuses mainly on professionals and organizations without considering the impact of the broader healthcare context such as healthcare regulation. The latter could however affect a just culture and initiatives to implement a just culture in healthcare organizations.

The objective of the current study is to explore the role governmental regulation has regarding a just culture in healthcare organizations, and to reflect on what this means for policy and practice of healthcare regulators.



For peer review only - http://bmjopen.bmj.com/site/about/guidelines.xhtml

# Methods

## Setting

Our study focuses on regulation of healthcare in the Netherlands. The role of the Dutch Health and Youth Care Inspectorate (from now on: inspectorate) is to supervise quality and safety of both healthcare organizations and individual healthcare professionals.[25, 26] The inspectorate uses two approaches: incident-based supervision following incidents and complaints, and risk-based supervision focusing on specific themes or type of providers. Dutch healthcare organizations are mandated by law to report sentinel events (meaning unintended harm to patients that led to death or serious injury) to the inspectorate and share the investigation report with the inspectorate.[27] In recent years, the inspectorate has focused its policy on learning and improvement of healthcare professionals and organizations, and in this context the current project takes place.

## Study design

Between 2017 and 2019, we studied how five healthcare organizations and a project group of the inspectorate worked on enhancing a just culture in healthcare organizations. The project underlying our study was initiated by the inspectorate with the aim of understanding what is needed for a just culture and how the inspectorate can contribute to this. For the project, researchers conducted a literature review with the objective of developing a working definition of just culture. This definition was not used as a normative framework but as a heuristic instrument to explore our empirical cases. Central elements in the working definition were openness about (a lack of) safety and fallibility, a balance between accountability and learning and improvement, considering different perspectives when an incident occurs, mutual trust between healthcare professionals and in relation to patients, and paying attention to what goes right in addition to what goes wrong. The complete working definition can be found in Appendix A.

We used qualitative research methods such as observations and interviews to explore experiences with working on a just culture and the relationship with regulation. Participating organizations (i.e. 3 mental healthcare providers and 2 hospitals) were recruited after a seminar of the inspectorate about just culture and each started their own project on working on a just culture. These projects varied from working on specific processes (e.g. incident investigations) to broader approaches on quality and safety policies in the organization. Simultaneously, a project group of the inspectorate held regular meetings to reflect on preliminary findings and their own role as inspectors in enabling a just culture. The goal of our study was to identify overarching themes related to the role of regulation in enabling a just culture.

## Data collection

In preparation of the empirical research in the organizations, interviews were held with employees of the inspectorate to gain insight into the way inspectors interpret the concept of a just culture and how they view their own role. Subsequently, we observed meetings, held interviews with healthcare professionals, managers and quality and safety officers, and conducted focus group interviews about working on a just culture and the role of regulation in


For peer review only - http://bmjopen.bmj.com/site/about/guidelines.xhtml

the participating organizations. Topic lists were developed and discussed by the research team to guide data collection. The interviews and focus groups were recorded (audio) and transcribed verbatim, while written reports were made of the observations. During the project, we presented and reflected on preliminary findings within the organizations. In addition, we organized three network meetings. Here, representatives of the five organizations and the inspectorate came together and shared their experiences to learn from each other. At the end of the project, we organized three focus groups with inspectors in which we fed back the results from the organizations and reflected on what these findings mean for the regulator. In total, the data collected for this study consisted of 61 interviews, 7 focus groups and 27 observations. Table 1 provides an overview of all data collection methods.

*Table 1: Overview of data collection*

| Location | Activities for data collection |
|---|---|
| Inspectorate | ○ 8 interviews with inspectors to explore the concept of just culture and potential role of the inspectorate at the start of the project<br>○ 3 focus groups with inspectors (4-8 per group) to reflect on the findings at the end of the project |
| Mental healthcare organisation #1 | ○ 7 dialogue sessions with ±4 participants of different layers of the organization in which participants discussed experiences and dilemmas in (working on) a just culture<br>○ 1 reflection session |
| Mental healthcare organisation #2 | ○ 2 dialogue sessions in which participants discussed experiences and dilemmas in (working on) a just culture<br>○ 10 interviews with participants of dialogue sessions<br>○ 6 interviews with professionals<br>○ 2 interviews with management |
| Mental healthcare organisation #3 | ○ 17 interviews<br>○ 4 observations<br>○ 2 focus groups<br>○ 1 reflection session |
| Hospital #1 | ○ 11 interviews with 14 persons<br>○ 2 focus groups |
| Hospital #2 | ○ 7 interviews<br>○ 12 observations<br>○ 1 conference meeting |
| Network sessions | ○ 3 meetings with organizations and inspectorate aimed at exchanging experiences between participating organizations |

## Data analysis

The analysis focused on exploring overarching and recurring themes in the data.[28] The first interviews were inductively coded by involved researchers in order to identify common


For peer review only - http://bmjopen.bmj.com/site/about/guidelines.xhtml

patterns in the data. These patterns included factors and mechanisms related to enabling a just culture in organizations in general, not on the role of regulation specifically. These patterns were then discussed, adjusted and further elaborated on during discussions within the research group, leading to a coding scheme. Subsequent transcripts of interviews and focus groups and notes of observations were coded using this scheme and new themes were added as they emerged. Findings from the transcripts, observations and meetings were discussed within the research group and fed back to participating organizations and inspectors throughout the project. To understand and reflect on the specific role of regulation in enabling a just culture, the findings were further analysed with the purpose of this study in mind. We primarily focused on perceptions and actual experiences with the impact of regulation on a just culture, yet also included respondent's perceptions of the potential role of regulation in enabling a just culture. Our analysis led to three main themes related to regulation and just culture.

### Patient and public involvement

There was no patient or public involvement.

### Research Ethics

The Medical Research Ethics Committee of the Erasmus Medical Centre determined that the study did not fall within the scope of the Medical Research Involving Human Subjects Act and as such did not require additional ethical approval (MEC-2018-054). All respondents were verbally informed about the study and gave their approval for recording interviews and focus groups. To ensure anonymity, some details from quotations have been adjusted and we do not specify from which organizations quotations come.

## Findings

From our analysis, we identified three themes that are important to understand the role of governmental regulation in enabling a just culture in healthcare organizations. The first concerns how regulation impacts a just culture in healthcare organizations. The second regards the relational and layered nature of a just culture. This extends beyond the role of regulation alone, yet in this study we focus on how it applies to regulation. The third theme entails specific challenges for regulators and inspectors when trying to enable a just culture in healthcare organizations.

### 1. Regulatory impact on a just culture

When respondents elaborated on the role and impact of the regulator in enabling a just culture, they referred to two important issues: the image of the regulator and the rigidity of forms and procedures.

#### *Police or driver of quality improvement?*

Respondents perceived the inspectorate as a threat for creating a just culture, as they come into play when things already have gone wrong to judge about what has gone and has been done

For peer review only

wrong. Although the inspectorate's scope and tasks are broader, this perception does affect the safety and openness that employees experience when trying to learn from incidents.

> *"That's how I see the inspectorate. When they come to the hospital, something is going on somewhere. They don't just show up, you know. The police also does not come to your house for a cup of coffee. Then there is something going on as well. That's how I see it."*

There is a perceived threat among professionals of being held responsible for (their share in) an incident, and that being open in their communications can backfire. Inspectors recognized this tension yet referred to the professional standards that healthcare professionals must adhere to. According to inspectors, not calling on individual responsibility is difficult when sentinel events involve culpable personal actions of a professional.

At the same time, the inspectorate may act as an important driver for quality improvement. The image of the inspectorate and possible measures they might take ensure that healthcare organizations take sentinel event investigations seriously. The involvement of the inspectorate makes healthcare organisations to want to do such investigations thoroughly and make time and resources available for it.

> *"And well, then we found out that the medication used is already off the market in various hospitals. You just go deeper, deeper and deeper because of that investigation. I just wonder if the mandated investigation [of the inspectorate] had not been there, would we have gone that deep?"*

The inspectorate's image thus not only has negative consequences, but also implies authority that leads to action in organizations to improve patient safety.

***Rigid forms and procedures when things have gone wrong***

Although the inspectorate might be a catalyst for thorough investigative processes, this does not directly mean that these processes also contribute to learning among healthcare professionals. Respondents indicated that the tight timetable with hard deadlines for investigating and reporting sentinel events, in combination with the length of such reports, frustrate openness and thus learning. It means that there is limited space to reflect, and although reflection should be part of the investigation, it is not always experienced as such. There is a risk that meeting the inspectorate's requirements gains priority over the learning process:

> *"There is a time limit of 8 weeks, then it takes time before you get [the report] back from the inspectorate. And you have a time limit in which [a sentinel event] must be reported. So, you cannot just think calmly whether or not to report [the event], whether to investigate."*

Respondents experienced that properly recording everything for the investigation is important. They perceive that what is written down on paper is more important than what exactly happened in practice. This 'paper-based reality', in which the focus is mainly on factual matters in combination with the formal language used in the report, is insufficiently in line with how



For peer review only - http://bmjopen.bmj.com/site/about/guidelines.xhtml

professionals perceive their work. Consequently, professionals sometimes feel distanced from the reporting.

> *"It is a business-like format that mainly looks at factual summaries of things that have been discussed. (...) People do perceive that [the inspectorate] finds it more important that everything on paper is correct instead of the actual care we provided. Because that story is almost deleted from those documents. (...) So, in the team you see that when the report is finally done, that people have a bit of a hangover from [the investigation report]."*

Some inspectors acknowledged the need for available instruments and procedures to match the goal of learning and improving and recognize that current forms and procedures do not facilitate such an approach.

> *"I also think that if we want to get a just culture into our DNA from our actions, then we need to carefully examine the systems we work with, the forms we work with and the questions we ask and see whether they are just culture-proof and are focused on learning and prevention in the future."*

This requires the inspectorate to reflect on their own procedures from such a learning perspective.

**2. The relational and layered nature of a just culture**

Just culture was considered relational and layered by respondents, meaning that relationships between actors from different layers within and outside the healthcare organization might hinder or promote a just culture. Two important aspects of this relational and layered nature were mentioned by respondents as relevant for enabling (or hindering) a just culture in organizations: relationships of mutual trust, and the role of publicity and legislation.

***Building relationships of mutual trust***

Respondents from the organizations reported that a just culture relies on mutual trust. This applies to different actors within the healthcare organization, for example between professionals, superiors and management, but also in relation to the inspectorate. This means that how employees experience interactions with the inspectorate and individual inspectors – and whether those are positive or negative – is important for experiencing a just culture. Their feeling during inspections is influenced by the procedures and correspondence of the inspectorate.

> *"You wait for some sort of grade from your schoolteacher, it always feels like that. While you would like much more dialogue at the table, 'what do we learn from this'? That the inspectorate thus gains a sense of the learning capacity of an organization from their supervisory role, and not through letters with reference numbers, on which we then disagree and what results in writing another letter back."*

Building trust is also about being open about regulatory procedures as an inspector, without perhaps always being able to offer a safe environment. This procedural clarity was seen by



For peer review only - http://bmjopen.bmj.com/site/about/guidelines.xhtml

inspectors as part of a just culture, in which the quality and safety of patient care must come first. It means that inspectors must be clear that they cannot guarantee that someone will not be held personally accountable in case of sentinel events but that it is very unlikely. Inspectors noticed that this openness and transparency about procedures on their part – in general or specifically when an incident has occurred – contributes to understanding and trust, and respondents from organizations experienced it positively when the inspectorate elaborated and explained their procedures and its position.

> *"Two inspectors visited the staff and indicated what their working method is and how they view our hospital. And that is quite enlightening. We perceive them as a very annoying organization that is trying to catch us, but in reality, it's not that bad. [Our staff] suddenly sees a face of these people, instead of just their firm notes and letters."*

Recognizing and building on the relationality of regulation is of importance in enabling a just culture as it helps in building trust and being able to talk about vulnerabilities.

***The role of publicity and legislation***

A factor that according to respondents influenced openness, and thus learning, is publicity. While openness about an incident or sentinel event within the team or organization was seen by respondents as an essential component of a just culture, external publication and publicity pose a threat to it. Healthcare professionals perceived that anything they say might become public at some point, either via reports of the inspectorate or via the media. And although the inspectorate does not publish investigation reports with names of professionals, some professionals mentioned that even anonymized data are easily traceable. This perceived threat sometimes leads to openness and learning being disrupted, whereby what is written down on paper is again seen as important. In addition to the perception that paperwork seems more important than learning, choosing what to write down and what not is also about hedging against any potential negative consequences of an investigation report. It could be more worthwhile to sit down and talk and reflect without writing things down on paper, as one of the healthcare professionals said:

> *"I think so, if we lock ourselves up in a room for one afternoon, then there would just come out more. Things that are not written on paper and that do not go to the [national newspaper]. We would then learn even more from [the sentinel event]."*

Inspectors recognized the disrupting influence of media and other external actors on a just culture and have to deal with such influences themselves too. They experienced that pressure from the media can lead to a feeling of insecurity among healthcare professionals.

In addition to potential publicity, respondents also mentioned the inhibiting nature of existing legislation and risks of litigation. Even though the inspectorate might adopt a learning perspective in their regulation, existing legislation on disciplinary complaints still focuses on individual accountability. Patients, for example, may choose to file a complaint against individual healthcare professionals.



For peer review only - http://bmjopen.bmj.com/site/about/guidelines.xhtml

> *"There is also the disciplinary judge who is breathing down your neck. We are talking about a just culture, but how open is it when the threat of litigation lingers in the background?"*

Whilst accounting for sentinel events was thus seen as enabling learning, professionals feared the publicity that might be involved.

**3. Challenges for regulators and inspectors in enabling a just culture**

Respondents, and specifically inspectors, mentioned several challenges for the regulator when trying to contribute to a just culture in healthcare organizations. These challenges related to the assessment of a just culture in practice, and the tension that can arise between informal contact of an inspector with an organization and formal measures that can be taken.

***Assessing a just culture***

Inspectors struggled with the question how to assess whether an organization has a just culture. Some inspectors indicated that instruments are available to get a feel for this, such as inspection frameworks on Trust and on Good Governance, in which openness, transparency and trust are important components. At the same time, inspectors realized that a just culture cannot be ticked off and that it is also about intuition and how confident you are that an organization itself is able to improve.

> *"Yes, a gut-feeling. When you are present at the administrative levels, then you need to understand the matter. So you ask the right questions to get a feeling of the organization. If that feeling is not good, then you should take a look at certain indicators."*

According to inspectors, asking questions implies a different attitude or style than a controlling one during an inspection visit. At the same time, as an inspector you never only act as a coach and discussion partner.

> *"We are also assigned a role depending on the situation. So, at one moment it is nice to be a discussion partner and at the other moment an organization needs you as a bogeyman to create urgency."*

The inspector cited above emphasizes the two roles inspectors can assume and that are expected of them, and the importance of finding a balance between giving space and keeping a firm hand on the healthcare provider. This means an inspector must be able to do both and must be able to sense which approach is necessary for a given situation.

***Informal contact and formal measures***

The fact that the inspectorate can impose sanctions can be at odds with the promotion of a just culture within healthcare organizations. According to inspectors, this makes it difficult because tensions arise between the space that an inspector sometimes wants to give to an organization to learn and improve (without formal interference from the inspectorate) and the policies and rules that prescribe certain sanctions, such as a monetary fine. Inspectors especially struggled

For peer review only - http://bmjopen.bmj.com/site/about/guidelines.xhtml

when they had really invested in the relationship with an organization to ensure that the organization takes responsibility for quality and safety.

> *"I am the contact person for this organization. I went through this whole process with them and they clearly learned. It feels wrong to give them a fine at the end of this when they have come up with solutions themselves. I think that in our cooperative relationship, which I understand is a special kind of relationship, that's not good."*

Individual inspectors sometimes give a bit of space to organizations because they feel that this contributes to learning within the organization. Yet, when formally judging an organization, which is also the inspectorate's task (including possible measures), the case is discussed within the inspectorate. Sometimes there are different perspectives on what should be done between the involved inspector and other inspectors, managers and the legal department. This makes it difficult, because for a healthcare organization it might seem as if there is a lot of space to learn and improve and figure things out whereas at the end of the process the organization might be confronted with an intervention from the inspectorate. So, although individual inspectors sometimes seem to have an eye for a just culture within the healthcare organization to facilitate learning, they are aware that trust in the relationship is fragile.

## Discussion

We explored the role of governmental regulation in enabling a just culture in healthcare organizations. Our results show that the regulator through its procedures and interaction with organizations has impact on learning processes and openness. Building mutual trust, for example by being clear about regulatory procedures and expectations, is deemed important, while publicity and external transparency might frustrate learning and openness. Our study moreover highlights challenges for regulators when it comes to assessing a just culture and the impact of legislation. We first provide a brief methodological reflection before we reflect on these findings.

### Strengths and limitations

A strength of this study is the amount and variety of collected data. This rich qualitative dataset from two healthcare sectors enables us to understand processes of just culture and regulation 'from within', which is needed as most of the literature on just culture is theoretical.[7, 29] The study has some limitations as well. First, healthcare organizations were recruited after a seminar of the inspectorate and as such were actively interested in working on a just culture with the inspectorate. It is unknown whether studying organizations that are less motivated or less comfortable with the inspectorate would have resulted in additional insights about the role of regulation in enabling a just culture. Second, we need to be careful to generalize these findings across and within settings as the precise role of regulation and regulators might depend on the context of a healthcare sector as well as the national context of regulation. Although the context of regulation will differ internationally, we do believe our findings are of international relevance as the mechanisms we discussed relate to previous findings about the role of regulation.[30, 31] How these mechanisms play out in each country might be different and could be input for future comparative research.


For peer review only - http://bmjopen.bmj.com/site/about/guidelines.xhtml

**Two issues for regulators when enabling a just culture**

For regulators, two issues seem important when aiming to enable a just culture. The first is the impact of regulatory procedures and actions on a just culture in healthcare organizations. As our study showed, the relation between the regulator and healthcare organizations, influences the space for openness, reflection and learning in healthcare organizations. It requires reflection from regulators on their policies and procedures, and an understanding of how they directly impact (either positively or negatively) the reflective space in organizations.[2, 32] A second issue is just culture as a topic of regulation itself. Inspectors felt the need to be able to assess whether an organization has a just culture. Although in international contexts tools have been developed to assess a just culture,[33] inspectors indicated that it requires intuition or a gut-feeling and that a just culture cannot simply be ticked off. It thus seems important that when choosing an assessment tool, it is used by inspectors to get a better understanding of an organization by combining it with forms of soft and hard intelligence, instead of directly actioning regulatory measures based on the outcomes of the assessment.[34, 35] The latter most likely will not lead to an organization working towards an open and learning culture but to an organization trying to score best on the measurements included in the assessment, risking to 'hit the target but miss the point'.[36]

**Balancing conflicting strategies**

Regulators are not an independent observer in monitoring quality and safety, but part of the healthcare playing field. Their actions and procedures influence practices within healthcare organizations. In our study this was apparent in the mentioned actions of inspectors through coaching or more policing strategies towards healthcare organizations. These strategies are inherent to responsive regulation theory describing persuasive and coercive enforcement approaches, and which assumes that regulators should start with persuasive strategies before considering coercive ones.[37] These strategies conflict and are not strictly successive as theory suggests, for example when expecting openness from professionals aimed at learning and at the same time keeping the possibility to file a formal complaint against an individual professional. The scope of the Dutch healthcare inspectorate – regulating both organizations and individual professionals – makes this conflict even more complex. At the same time, adopting different strategies makes that healthcare organizations take inspectors seriously, as they can adjust to the specific needs of an organization. For inspectors, it is important to communicate the intentions of regulation and to continuously reflect on how to balance these strategies in practice.[38] A further question for regulators would then be whether all inspectors should be able to conduct both strategies or whether these styles are represented by different inspectors. The former would require different and for some inspectors new skills, whereas the latter would possibly create tensions between inspectors focused on learning and inspectors focused on policing.[39]

**Beyond the vacuum: taking third parties into account**

A just culture requires psychological safety in healthcare organizations, but also in the relation between regulator and healthcare organization.[40, 41] However, this relation does not exist in a vacuum, and in enabling a just culture this is especially problematic when things have gone


For peer review only - http://bmjopen.bmj.com/site/about/guidelines.xhtml

wrong. Often, patients and patient bodies, politics and media, quite understandably, demand thorough investigations, partly substantiated by a concern that certain things will otherwise be kept under the table. We've seen many examples in the past where these concerns were warranted.[42] The involvement of these other parties also means that the incident and subsequent investigation is taken outside the relation of regulator and healthcare organization, and the publicity and attention influences openness and learning within the organization. [43] This is something inspectors are aware of and that poses an additional challenge when trying to enable a just culture as a regulator. Being transparent about regulatory procedures and intentions towards those other parties might contribute to lowering the temperature of heated public discussions and as such contributes to the psychological safety of those involved. For healthcare organizations, directly involving patients or their representatives might contribute to trust and being able to investigate and learn out of the public spotlight.[44]

## Conclusion

Regulators can have an important influence on a just culture in healthcare organizations. This means that when implementing just culture initiatives in healthcare organizations, the role and impact of regulation should be taken into account. For regulators to be able to contribute to a just culture, we recommend that they 1) become aware of the impact regulation and other stakeholders and policies have on a just culture, 2) adopt regulatory procedures that support reflection and learning in organizations, and 3) continuously reflect on how to balance coaching and policing strategies as inspectors. By doing so, regulators can contribute to learning within healthcare and as such improve quality and patient safety.

## Contributorship statement

The study was designed by IW, LH, GW and RB. Data was collected and analyzed by JWW, IW, LH, EvB, IL, GW and RB. A first draft of the paper was written by JWW. All authors contributed to and approved the final manuscript.

## Competing interests

None declared.

## Funding

The study was funded by the Netherlands Organisation for Health Research and Development (ZonMw), project number 516004613.

## Data sharing statement

No additional data are available.

## Ethics approval statement

The Medical Research Ethics Committee of the Erasmus Medical Centre determined that the study did not fall within the scope of the Medical Research Involving Human Subjects Act and as such did not require additional ethical approval (MEC-2018-054).



For peer review only - http://bmjopen.bmj.com/site/about/guidelines.xhtml

For peer review only

# References

1. Leistikow I. The three stages of safety improvement. *The BMJ Opinion* blog. January 18, 2019, 2019.
2. Wiig S, Aase K, Bal R. Reflexive spaces: Leveraging resilience into healthcare regulation and management. *Journal of patient safety*. 2021;
3. Wears RL. Standardisation and its discontents. *Cognition, technology & work*. 2015;17(1):89-94.
4. Peerally MF, Carr S, Waring J, Dixon-Woods M. The problem with root cause analysis. *BMJ quality & safety*. 2017;26(5):417-422.
5. Dekker S. *Just culture: Balancing safety and accountability*. CRC Press; 2016.
6. Reason J. *Human error*. Cambridge university press; 1990.
7. Dekker SW, Breakey H. 'Just culture:'Improving safety by achieving substantive, procedural and restorative justice. *Safety science*. 2016;85:187-193.
8. Kaur M, De Boer RJ, Oates A, Rafferty J, Dekker S. Restorative just culture: a study of the practical and economic effects of implementing restorative justice in an NHS trust. EDP Sciences; 2019:01007.
9. White RM, Delacroix R. Second victim phenomenon: Is 'just culture'a reality? An integrative review. *Applied Nursing Research*. 2020;56:151319.
10. Sharpe VA. Promoting patient safety: an ethical basis for policy deliberation. *The Hastings Center Report*. 2003;33(5):S3.
11. Frankel AS, Leonard MW, Denham CR. Fair and just culture, team behavior, and leadership engagement: The tools to achieve high reliability. *Health services research*. 2006;41(4p2):1690-1709.
12. Boysen PG, 2nd. Just culture: a foundation for balanced accountability and patient safety. *Ochsner J*. Fall 2013;13(3):400-6.
13. Khatri N, Brown GD, Hicks LL. From a blame culture to a just culture in health care. *Health Care Manage Rev*. Oct-Dec 2009;34(4):312-22. doi:10.1097/HMR.0b013e3181a3b709
14. Small D, Small RM, Green A. Improving safety by developing trust with a just culture. *Nurs Manag (Harrow)*. Apr 7 2022;29(2):32-41. doi:10.7748/nm.2021.e2030
15. Barkell NP, Snyder SS. Just culture in healthcare: An integrative review. *Nurs Forum*. Jan 2021;56(1):103-111. doi:10.1111/nuf.12525
16. David DS. The Association Between Organizational Culture and the Ability to Benefit From "Just Culture" Training. *J Patient Saf*. Mar 2019;15(1):e3-e7. doi:10.1097/pts.0000000000000561
17. Vogelsmeier A, Scott-Cawiezell J, Miller B, Griffith S. Influencing leadership perceptions of patient safety through just culture training. *J Nurs Care Qual*. Oct-Dec 2010;25(4):288-94. doi:10.1097/NCQ.0b013e3181d8e0f2
18. Petschonek S, Burlison J, Cross C, et al. Development of the just culture assessment tool: measuring the perceptions of health-care professionals in hospitals. *J Patient Saf*. Dec 2013;9(4):190-7. doi:10.1097/PTS.0b013e31828fff34
19. Freeman M, Morrow LA, Cameron M, McCullough K. Implementing a Just Culture: Perceptions of Nurse Managers of Required Knowledge, Skills and Attitudes. *Nurs Leadersh (Tor Ont)*. 2016;29(4):35-45. doi:10.12927/cjnl.2016.24985
20. White RM, Delacroix R. Second victim phenomenon: Is 'just culture' a reality? An integrative review. *Appl Nurs Res*. Dec 2020;56:151319. doi:10.1016/j.apnr.2020.151319
21. Marx D. Patient Safety and the Just Culture. *Obstetrics and gynecology clinics of North America*. 2019;46(2):239-245.
22. Weiner BJ, Hobgood C, Lewis MA. The meaning of justice in safety incident reporting. *Social science & medicine*. 2008;66(2):403-413.

For peer review only

23. Burhans LD, Chastain K, George JL. Just Culture and Nursing Regulation: Learning to Improve Patient Safety. *Journal of Nursing Regulation*. 2012/01/01/ 2012;2(4):43-49. doi:https://doi.org/10.1016/S2155-8256(15)30254-4
24. Marx DA. *Patient safety and the" just culture": a primer for health care executives*. Trustees of Columbia University; 2001.
25. Hout F, Nienhuis E, Robben P, Frederiks B. Supervision by the Dutch healthcare inspectorate. *Eur J Health L*. 2010;17:347.
26. Weenink J-W, Wallenburg I, Leistikow I, Bal RA. Publication of inspection frameworks: a qualitative study exploring the impact on quality improvement and regulation in three healthcare settings. *BMJ Quality & Safety*. 2021;30(10):804-811.
27. de Kam D, Kok J, Grit K, Leistikow I, Vlemminx M, Bal R. How incident reporting systems can stimulate social and participative learning: A mixed-methods study. *Health Policy*. 2020;124(8):834-841.
28. Braun V, Clarke V. *Successful qualitative research: A practical guide for beginners*. sage; 2013.
29. O'Donovan R, Ward M, De Brún A, McAuliffe E. Safety culture in health care teams: A narrative review of the literature. *Journal of Nursing Management*. 2019;27(5):871-883.
30. Bardach E, Kagan RA, Fund TC. *Going by the Book: The Problem of Regulatory Unreasonableness*. Temple University Press; 1982.
31. Rutz S, Mathew D, Robben P, de Bont A. Enhancing responsiveness and consistency: Comparing the collective use of discretion and discretionary room at inspectorates in E ngland and the N etherlands. *Regulation & Governance*. 2017;11(1):81-94.
32. Kok J, de Kam D, Leistikow I, Grit K, Bal R. Epistemic Injustice in Incident Investigations: A Qualitative Study. *Health Care Analysis*. 2022/05/31 2022;doi:10.1007/s10728-022-00447-3
33. Petschonek S, Burlison J, Cross C, et al. Development of the Just Culture Assessment Tool (JCAT): Measuring the perceptions of healthcare professionals in hospitals. *Journal of patient safety*. 2013;9(4):190.
34. Kok J, Wallenburg I, Leistikow I, Bal R. The doctor was rude, the toilets are dirty. Utilizing 'soft signals' in the regulation of patient safety. *Safety Science*. 2020;131:104914.
35. Martin GP, McKee L, Dixon-Woods M. Beyond metrics? Utilizing 'soft intelligence' for healthcare quality and safety. *Social Science & Medicine*. 2015;142:19-26.
36. Radnor Z. Hitting the target and missing the point? Developing an understanding of organizational gaming. *Performance Information in the Public Sector*. Springer; 2008:94-105.
37. Ayres I, Braithwaite J. *Responsive regulation: Transcending the deregulation debate*. Oxford University Press, USA; 1992.
38. Mascini P, Wijk EV. Responsive regulation at the Dutch food and consumer product safety authority: An empirical assessment of assumptions underlying the theory. *Regulation & Governance*. 2009;3(1):27-47.
39. Furnival J, Walshe K, Boaden R. Emerging hybridity: comparing UK healthcare regulatory arrangements. *Journal of health organization and management*. 2017;
40. Khatri N, Brown GD, Hicks LL. From a blame culture to a just culture in health care. *Health care management review*. 2009;34(4):312-322.
41. Groeneweg J, Ter Mors E, Van Leeuwen E, Komen S. The long and winding road to a just culture. OnePetro; 2018:
42. Leape LL. A Conspiracy of Silence: Disclosure, Apology, and Restitution. *Making Healthcare Safe*. Springer; 2021:293-317.
43. Kok J. A standard story: On the use and consequences of standards in healthcare regulation. 2021;

For peer review only

44. Bouwman R, De Graaff B, De Beurs D, Van de Bovenkamp H, Leistikow I, Friele R. Involving patients and families in the analysis of suicides, suicide attempts, and other sentinel events in mental healthcare: a qualitative study in the Netherlands. *International journal of environmental research and public health*. 2018;15(6):1104.

For peer review only



For peer review only - http://bmjopen.bmj.com/site/about/guidelines.xhtml

**Attachment A**

**Working definition of just culture in the project**

A just culture is a culture of trust in which employees feel free to be open about insecurity and fallibility. In particular, the focus is on the behavior rather than the outcomes of that behavior, because behavior is something that healthcare professionals can control, while outcomes are partly dependent on factors outside the direct sphere of influence of the healthcare professional.

Learning and improving instead of punishment are paramount in a just culture. There is an open, safe and informal culture of approach, in which people work together on the quality of care in a continuous learning cycle. However, this does not mean that people within a just culture cannot be held responsible for their behavior. A distinction is made between 'blaming and being punished' and 'accountability'.

People who are involved in undesirable outcomes or who have made mistakes are treated fairly and a balance is struck between accountability for a mistake made and learning and improving from that mistake. A mistake made is mainly seen as a reason to learn from within a just culture. System factors are also explicitly considered. The relevant question is: what caused this situation to arise? Peer support of 'second victims' is a natural part of a just culture, in addition to, of course, attention for primary victims.

A just culture recognizes that there is no single truth of an event. It is about valuing and considering multiple perspectives on an event. Justice means: do not judge from one perspective, but include as many perspectives as possible. This plurality requires a dialogue. Norms, such as those laid down in clinical guidelines, are considered here, but never used as a standard; attention is always paid to the situation from which action was taken and the interpretations of the standard that were used.

A central concept for a just culture is trust; trust among employees so they can also speak out to each other, trust of employees in managers that they are treated fairly, trust of patients and their families that they are treated with respect and that errors result in learning and improvement.

For peer review only

# Standards for Reporting Qualitative Research (SRQR)*

http://www.equator-network.org/reporting-guidelines/srqr/

**Page/line no(s).**

## Title and abstract

| | |
|---|---|
| **Title** - Concise description of the nature and topic of the study Identifying the study as qualitative or indicating the approach (e.g., ethnography, grounded theory) or data collection methods (e.g., interview, focus group) is recommended | Separate in manuscript central |
| **Abstract** - Summary of key elements of the study using the abstract format of the intended publication; typically includes background, purpose, methods, results, and conclusions | Separate in manuscript central |

## Introduction

| | |
|---|---|
| **Problem formulation** - Description and significance of the problem/phenomenon studied; review of relevant theory and empirical work; problem statement | P1 |
| **Purpose or research question** - Purpose of the study and specific objectives or questions | P1/L24 |

## Methods

| | |
|---|---|
| **Qualitative approach and research paradigm** - Qualitative approach (e.g., ethnography, grounded theory, case study, phenomenology, narrative research) and guiding theory if appropriate; identifying the research paradigm (e.g., postpositivist, constructivist/ interpretivist) is also recommended; rationale** | P3 |
| **Researcher characteristics and reflexivity** - Researchers' characteristics that may influence the research, including personal attributes, qualifications/experience, relationship with participants, assumptions, and/or presuppositions; potential or actual interaction between researchers' characteristics and the research questions, approach, methods, results, and/or transferability | - |
| **Context** - Setting/site and salient contextual factors; rationale** | P2 |
| **Sampling strategy** - How and why research participants, documents, or events were selected; criteria for deciding when no further sampling was necessary (e.g., sampling saturation); rationale** | P2-3 |
| **Ethical issues pertaining to human subjects** - Documentation of approval by an appropriate ethics review board and participant consent, or explanation for lack thereof; other confidentiality and data security issues | P4/L17 |
| **Data collection methods** - Types of data collected; details of data collection procedures including (as appropriate) start and stop dates of data collection and analysis, iterative process, triangulation of sources/methods, and modification of procedures in response to evolving study findings; rationale** | P3/L2 |



For peer review only - http://bmjopen.bmj.com/site/about/guidelines.xhtml

| | |
|---|---|
| **Data collection instruments and technologies** - Description of instruments (e.g., interview guides, questionnaires) and devices (e.g., audio recorders) used for data collection; if/how the instrument(s) changed over the course of the study | P3/L5 |
| **Units of study** - Number and relevant characteristics of participants, documents, or events included in the study; level of participation (could be reported in results) | P3 |
| **Data processing** - Methods for processing data prior to and during analysis, including transcription, data entry, data management and security, verification of data integrity, data coding, and anonymization/de-identification of excerpts | P3/L6 |
| **Data analysis** - Process by which inferences, themes, etc., were identified and developed, including the researchers involved in data analysis; usually references a specific paradigm or approach; rationale** | P4/L3 |
| **Techniques to enhance trustworthiness** - Techniques to enhance trustworthiness and credibility of data analysis (e.g., member checking, audit trail, triangulation); rationale** | P4/L13 |

**Results/findings**

| | |
|---|---|
| **Synthesis and interpretation** - Main findings (e.g., interpretations, inferences, and themes); might include development of a theory or model, or integration with prior research or theory | P4/L25 |
| **Links to empirical data** - Evidence (e.g., quotes, field notes, text excerpts, photographs) to substantiate analytic findings | P4/L41 |

**Discussion**

| | |
|---|---|
| **Integration with prior work, implications, transferability, and contribution(s) to the field -** Short summary of main findings; explanation of how findings and conclusions connect to, support, elaborate on, or challenge conclusions of earlier scholarship; discussion of scope of application/generalizability; identification of unique contribution(s) to scholarship in a discipline or field | P9/L5 |
| **Limitations** - Trustworthiness and limitations of findings | P9/L15 |

**Other**

| | |
|---|---|
| **Conflicts of interest** - Potential sources of influence or perceived influence on study conduct and conclusions; how these were managed | Not in manuscript but separate statement in submission process |
| **Funding** - Sources of funding and other support; role of funders in data collection, interpretation, and reporting | Not in manuscript but separate statement in submission process |



For peer review only - http://bmjopen.bmj.com/site/about/guidelines.xhtml

For peer review only

*The authors created the SRQR by searching the literature to identify guidelines, reporting standards, and critical appraisal criteria for qualitative research; reviewing the reference lists of retrieved sources; and contacting experts to gain feedback. The SRQR aims to improve the transparency of all aspects of qualitative research by providing clear standards for reporting qualitative research.

**The rationale should briefly discuss the justification for choosing that theory, approach, method, or technique rather than other options available, the assumptions and limitations implicit in those choices, and how those choices influence study conclusions and transferability. As appropriate, the rationale for several items might be discussed together.

**Reference:**
O'Brien BC, Harris IB, Beckman TJ, Reed DA, Cook DA. **Standards for reporting qualitative research: a synthesis of recommendations.** *Academic Medicine*, Vol. 89, No. 9 / Sept 2014 DOI: 10.1097/ACM.0000000000000388

For peer review only
